# Cost‐Effectiveness of Pembrolizumab Plus Trastuzumab and Chemotherapy Versus Trastuzumab Plus Chemotherapy as First‐Line Treatment of HER2‐Positive Gastric or Gastroesophageal Junction Adenocarcinoma in China

**DOI:** 10.1002/cam4.71379

**Published:** 2025-11-20

**Authors:** Yifang Liang, Yuyanzi Zhang, Hongfei Hu, Yan Li, Aixia Ma, Xin Guan

**Affiliations:** ^1^ School of International Pharmaceutical Business China Pharmaceutical University Nanjing Jiangsu Province China; ^2^ Center for Pharmacoeconomics and Outcomes Research China Pharmaceutical University Nanjing Jiangsu Province China

**Keywords:** China, cost‐effectiveness analysis, HER2‐positive gastric cancer, pembrolizumab

## Abstract

**Objective:**

This study aimed to evaluate the cost‐effectiveness of pembrolizumab in combination with trastuzumab and chemotherapy (PEM + TRAS + Chemo) as a first‐line treatment for patients with advanced HER2‐positive gastric or gastroesophageal junction (GEJ) adenocarcinoma from the perspective of the Chinese healthcare system.

**Methods:**

Clinical data from the KEYNOTE‐811 trial were used to develop a partitioned survival model for HER2‐positive gastric or GEJ adenocarcinoma. The model estimated quality‐adjusted life years (QALYs), life years (LYs), and total lifetime costs. The primary outcome was the Incremental Cost‐Effectiveness Ratio (ICER), reflecting the cost per additional QALY. Only direct medical costs were considered, with drug prices sourced from the China Drug Bidding Database and other costs and utility values derived from published literature. Uncertainty analyses were conducted to test the robustness of the model, and subgroup analyses were performed to assess cost‐effectiveness in different patient populations.

**Results:**

In the base‐case analysis, the PEM + TRAS + Chemo regimen increased LYs by 0.17 and QALYs by 0.19, at an additional cost of $9874.08, resulting in an ICER of $53,160.95/QALY, which exceeds three times the per capita GDP of China ($39,999.86). Subgroup analysis based on the patient's programmed death‐ligand 1 (PD‐L1) combined positive score (CPS) showed that for PD‐L1 (CPS ≥ 1) patients, the ICER was $49,849.43 per QALY. Uncertainty analysis indicated that the proportion of patients receiving subsequent systemic treatment had the most significant impact on the model results.

**Conclusion:**

In China, for first‐line treatment of HER2‐positive advanced gastric or GEJ adenocarcinoma, the PEM + TRAS + Chemo regimen is not cost‐effective when compared to the TRAS + Chemo regimen, unless the price of pembrolizumab is reduced.

## 1 Introduction

Gastric cancer (GC) represents a significant global health burden, with over 968,000 new cases and approximately 660,000 deaths reported in 2022. Gastric cancer ranks as the fifth most common cancer worldwide in terms of both incidence and mortality, with particularly high prevalence in East Asia and Eastern Europe [1]. Notably, in 2022, GC was among the top five causes of cancer‐related mortality for both sexes in China, with crude mortality rates of 25.18/100,000 for males and 11.41/100,000 for females. This malignancy emerged as the leading cause of cancer‐related mortality in several western regions of China, including Gansu, Qinghai, and the Tibet Autonomous Region [2, 3]. The nonspecific clinical manifestations of early gastric cancer often lead to delayed diagnosis, with many patients presenting at advanced stages when symptoms become more clinically apparent [4]. The human epidermal growth factor receptor 2 (HER2)‐positive subtype constitutes a significant molecular classification of gastric cancer, representing approximately 20% of all diagnosed cases [5]. The therapeutic o ptions for this patient population remain limited, especially regarding first‐line treatments [6]. The recommended first‐line regimen for HER2‐positive gastric or gastroesophageal junction (GEJ) adenocarcinoma involves trastuzumab combined with either capecitabine plus oxaliplatin or fluorouracil plus cisplatin [7, 8]. Despite widespread adoption of trastuzumab as first‐line therapy for advanced HER2‐positive gastric cancer, its clinical efficacy is still limited by poor prognosis and the development of therapeutic resistance [9].

In recent years, the introduction of immune checkpoint inhibitors has expanded treatment options for these patients [10]. Pembrolizumab, a humanized monoclonal antibody targeting programmed cell death protein 1 (PD‐1), has shown substantial clinical efficacy in treating various malignancies. The phase III KEYNOTE‐062 trial demonstrated that combining pembrolizumab and chemotherapy enhances the anti‐tumor immune response and improves survival outcomes [11]. Furthermore, the phase III KEYNOTE‐590 trial confirmed that pembrolizumab combined with chemotherapy significantly improves both overall survival (OS) and progression‐free survival (PFS) in advanced esophageal adenocarcinoma and Siewert type I gastroesophageal junction cancer, compared to chemotherapy alone, with an acceptable safety profile [12]. The phase III KEYNOTE‐811 trial found that incorporating pembrolizumab into first‐line therapy with trastuzumab and fluoropyrimidine‐platinum chemotherapy significantly extends PFS and improves objective response rates [13, 14]. This therapeutic benefit is particularly evident in HER2‐positive gastroesophageal adenocarcinoma patients, with the PD‐L1‐positive subgroup experiencing the greatest clinical benefit. Mechanistically, pembrolizumab enhances tumor‐specific immune responses, leading to improved overall survival, particularly in PD‐L1‐positive patients, thereby underscoring the therapeutic synergy between immune checkpoint inhibition and HER2‐targeted therapy [15, 16, 17]. Despite promising initial outcomes suggesting the potential of pembrolizumab in combination with trastuzumab and chemotherapy, a comprehensive evaluation of its cost‐effectiveness is still lacking. This study aims to bridge this gap by evaluating the cost‐effectiveness of pembrolizumab in combination with trastuzumab and chemotherapy (PEM + TRAS + Chemo) compared to trastuzumab plus chemotherapy (TRAS + Chemo) as a first‐line treatment for patients with advanced HER2‐positive gastric or GEJ adenocarcinoma. The analysis was conducted from the perspective of the Chinese healthcare system by estimating the incremental cost‐effectiveness ratio (ICER) of the combination regimen.

## 2 Method

### 2.1 Population and Interventions

The patient demographics and treatment protocols were derived from the KEYNOTE‐811 trial [13]. Eligibility criteria required participants to be ≥ 18 years of age with a diagnosis of previously untreated, unresectable, or metastatic gastric or gastroesophageal junction adenocarcinoma, confirmed by pathological evidence of HER2 positivity. HER2 positivity was defined as an immunohistochemistry score of 3+ or 2+, combined with positive in situ hybridization or fluorescence in situ hybridization. Patients were required to have measurable disease according to RECIST v1.1, an Eastern Cooperative Oncology Group (ECOG) performance status of 0–1, a life expectancy > 6 months, adequate organ function, and sufficient tumor samples for assessing PD‐L1 and microsatellite instability status. A subgroup was defined by a PD‐L1 combined positive score (CPS) ≥ 1.

A total of 698 patients were randomly assigned to receive either pembrolizumab (200 mg) or placebo via intravenous infusion every 3 weeks (Q3W). All patients also received trastuzumab 6 mg/kg intravenously Q3W, following a loading dose of 8 mg/kg. Patients were treated with one of two chemotherapy regimens: fluorouracil 800 mg/m^2^ intravenously on days 1–5 plus cisplatin 80 mg/m^2^ Q3W, or capecitabine 1000 mg/m^2^ orally twice daily on days 1–14 and oxaliplatin 130 mg/m^2^ Q3W. Treatment continued for up to 35 cycles until disease progression, unacceptable toxicity, investigator discretion, or patient withdrawal. According to the Chinese Society of Clinical Oncology (CSCO) Gastric Cancer Guidelines (2023), the recommended second‐line therapy is a combination of ramucirumab and paclitaxel [18]. It was assumed that all patients requiring second‐line therapy would receive this regimen. The regimen consists of ramucirumab (8 mg/kg intravenously on days 1 and 15) combined with paclitaxel (80 mg/m^2^ intravenously on days 1, 8, and 15) in a 28‐day cycle.

### 2.2 Model Structure

**FIGURE 1:**
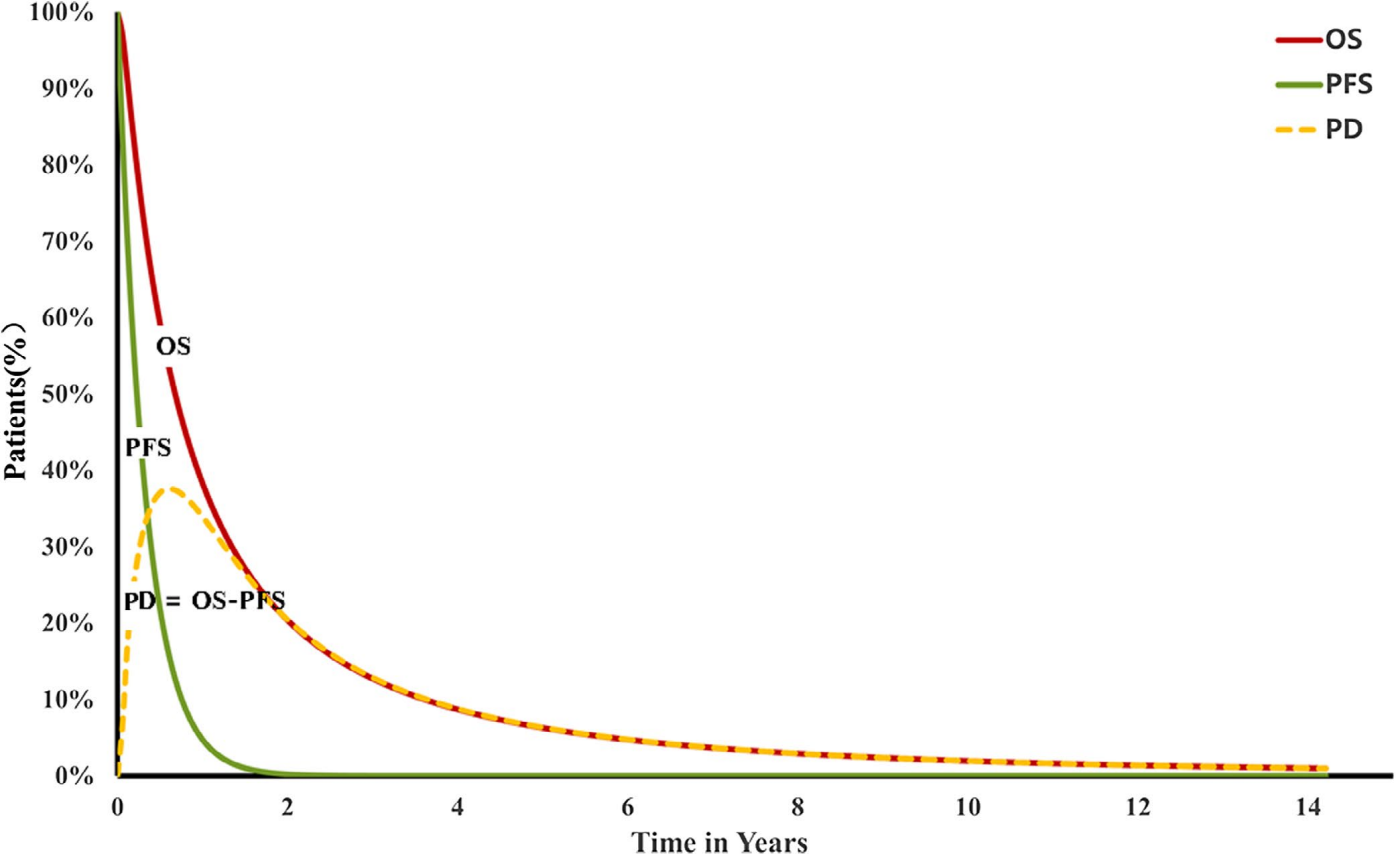
The model structure of PSM. OS, overall survival; PD, progressive disease; PFS, progression‐free survival; PSM, partitioned survival model.

This study developed a partitioned survival model (PSM) using Microsoft Excel to evaluate disease progression in patients with advanced HER2‐positive gastric or GEJ adenocarcinoma undergoing first‐line treatment. The model incorporates two survival curves (PFS and OS) to define three mutually exclusive health states: PFS, progressive disease (PD), and death (Figure 1). Patients entered the model in the PFS state and could either remain stable or transition to more advanced states, with no possibility of regression. The time horizon extended over patients' lifetime, with 3‐week cycles aligned with the dosing schedules of both treatment arms. Half‐cycle corrections were applied, assuming state transitions occurred at mid‐cycle. Key outcomes included total costs, life years (LYs), quality‐adjusted life years (QALYs), and ICER between regimens. In accordance with the Chinese Pharmacoeconomic Evaluation Guidelines, a 5% annual discount rate was applied to both future costs and outcomes [19]. All costs were adjusted to 2024 values using local Consumer Price Index data and converted to US dollars based on an exchange rate of $1 = ¥7.1812 [20].

### 2.3 Clinical Data Inputs

**FIGURE 2:**
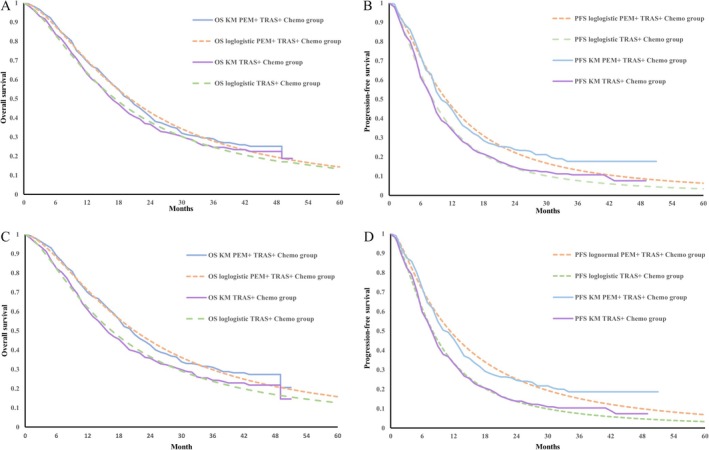
Kaplan–Meier and Fitted Survival Curves for Overall Survival and Progression‐Free Survival. (A) Kaplan–Meier and fitted curves for overall survival (OS) in the total population. (B) Kaplan–Meier and fitted curves for progression‐free survival (PFS) in the total population. (C) Kaplan–Meier and fitted curves for OS in patients with a PD‐L1 combined positive score (CPS) ≥ 1. (D) Kaplan–Meier and fitted curves for PFS in patients with PD‐L1 CPS ≥ 1. PEM + TRAS + Chemo: Pembrolizumab plus trastuzumab and chemotherapy; TRAS + Chemo: Trastuzumab plus chemotherapy; PD‐L1 CPS ≥ 1: PD‐L1 combined positive score of 1 or higher.

The clinical data used in this study were obtained from the KEYNOTE‐811 trial [13]. Long‐term survival data were generated through parameter distribution fitting and extrapolation of PFS and OS curves derived from this clinical trial. Data points were extracted from the original survival curves using GetData Graph Digitizer software (version 2.25). Subsequently, R statistical software (version 4.4.2) was used to reconstruct patient‐level data and perform parameter distribution fitting, which included exponential, Gamma, Gompertz, Weibull, log‐logistic, and log‐normal distributions. Model selection was based on visual inspection combined with statistical criteria, including the Akaike Information Criterion (AIC) and Bayesian Information Criterion (BIC), with lower values indicating a superior model fit. The fitting results are presented in Tables S2–S11, and the corresponding fitted and extrapolated curves are shown in Figure 2. To validate the selected models, their outputs were compared with the original KEYNOTE‐811 trial data. The results confirmed a strong concordance between the model‐estimated and trial‐reported outcomes. Specifically, in the overall population's pembrolizumab arm, the model‐estimated median OS was identical to the trial's at 20.0 months, while the median PFS was closely matched at 9.84 months versus 10.0 months, both with largely overlapping 95% confidence intervals. This high degree of accuracy extended to the PD‐L1 CPS ≥ 1 subgroup and the placebo group, where the differences for all key endpoints were less than 0.1 months. This strong concordance validates the model's reliability and supports its suitability for extrapolation.

### 2.4 Inputs of Cost and Utility

This study was conducted from the perspective of the Chinese healthcare system, focusing solely on direct medical costs, which include drugs, administration, follow‐up, adverse events (AEs), and end‐of‐life costs. Drug costs were obtained from the median prices of bid‐winning products listed in the China Drug Bidding Database (https://www.menet.com.cn/). Other expenses were based on previously published research reports and relevant literature. The frequency of follow‐up visits was determined according to the CSCO Guidelines and expert consultations. The associated costs for these visits—which included electrocardiograms, CT scans, tumor marker tests, blood biochemistry tests, blood tests, and liver and kidney function tests—were collected from healthcare documents in Guangxi Province [21]. To simplify the model, only severe AEs (Grade ≥ 3) with an incidence rate of ≥ 5% were considered. The incidence rates of these severe AEs were derived from the KEYNOTE‐811 clinical trial. The cost associated with each AE was calculated by multiplying the incidence rate of each AE by the management cost per event occurrence. It was assumed that all AEs occurred within the first cycle and only once during treatment. The drug cost per cycle was calculated by multiplying the unit cost of the drug by the dosage administered per cycle. This study follows the guidelines set by the National Healthcare Security Administration (NHSA), assuming a patient's body surface area is 1.6 m^2^ and weight is 60 kg to simplify drug dosage calculations and cost estimations. Given that the “Life Key—Cancer Immunotherapy Patient Assistance Program” offers patients an assistance plan for pembrolizumab, this study incorporated this assistance program into the model [22].

This study employed utility values ranging from 0 to 1 to assess the quality of life associated with various health states. In the absence of explicit utility value data from the KEYNOTE‐811 clinical trial, relevant utility values were derived from previously published literature. Specifically, the PFS utility values used in this study were reported in prior studies, based on EQ‐5D responses collected from patients with advanced gastric cancer in the ToGA trial, and calculated using the Japanese scoring algorithm [23]. Since the ToGA trial did not collect EQ‐5D data for post‐progression states, a post‐progression utility value of 0.577 was adopted from NICE Technology Appraisal 208, which was based on a previous health technology assessment of sunitinib for gastrointestinal stromal tumors and deemed appropriate for similar patient populations with advanced cancer [23, 24]. Additionally, this study incorporated the negative utility of severe AEs, with specific parameters and distributions outlined in Table 1.

**TABLE 1:** The parameters input of the model.

Parameters	Baseline	Range		Distribution	References
		Minimum	Maximum		
Cost ($)					
Follow‐up per cycle	45.01	36.01	54.02	Gamma	Health care document
Administration per cycle	62.40	49.92	74.89	Gamma	[25]
End‐of‐life	2122.47	1697.97	2546.96	Gamma	[25]
Best supportive care	348.00	278.40	417.61	Gamma	[26]
Cost of drugs					
Pembrolizumab/100 mg	2495.13	1996.10	2495.13	Gamma	menet.com.cn
Trastuzumab/1 mg	1.74	1.39	2.09	Gamma	menet.com.cn
Fluorouracil/100 mg	1.80	1.44	2.16	Gamma	menet.com.cn
Cisplatin/10 mg	1.19	0.95	1.43	Gamma	menet.com.cn
Capecitabine/1000 mg	0.59	0.47	0.71	Gamma	menet.com.cn
Oxaliplatin/100 mg	57.98	46.38	69.57	Gamma	menet.com.cn
Paclitaxel/10 mg	2.58	2.07	3.10	Gamma	menet.com.cn
Ramucirumab/1 mg	6.09	4.87	7.31	Gamma	menet.com.cn
Cost of serious adverse events					
Neutropenia	551.40	441.12	661.68	Gamma	[27]
Platelet count decreased	1648.55	1318.84	1978.26	Gamma	[27]
Anemia	486.62	389.30	583.95	Gamma	[27]
Diarrhea	46.01	36.81	55.21	Gamma	[27]
Neutrophil count decreased	551.40	441.12	661.68	Gamma	[27]
Risk of serious adverse events in PEM + TRAS + Chemo group (%)					
Neutropenia	6.29%	5.03%	7.54%	Beta	[13]
Platelet count decreased	6.29%	5.03%	7.54%	Beta	[13]
Anemia	6.00%	4.80%	7.20%	Beta	[13]
Diarrhea	8.86%	7.09%	10.63%	Beta	[13]
Neutrophil count decreased	8.00%	6.40%	9.60%	Beta	[13]
Risk of serious adverse events in TRAS + Chemo group (%)					
Platelet count decreased	6.65%	5.32%	7.98%	Beta	[13]
Anemia	5.78%	4.62%	6.94%	Beta	[13]
Diarrhea	7.80%	6.24%	9.36%	Beta	[13]
Neutrophil count decreased	8.67%	6.94%	10.40%	Beta	[13]
Utility					
PFS	0.797	0.598	0.966	Beta	[23]
PD	0.577	0.433	0.721	Beta	[23, 24]
Neutropenia	0.39	0.31	0.47	Gamma	[28]
Platelet count decreased	0.11	0.08	0.14	Gamma	[27]
Anemia	0.07	0.05	0.09	Gamma	[27]
Diarrhea	0.04	0.03	0.05	Gamma	[28]
Neutrophil count decreased	0.2	0.15	0.25	Gamma	[27]
Discount rate	0.05	0.00	0.08	Fixed	[19]
Body weight (kg)	60.00	48	72	Fixed	NHSA's guidelines
Body surface area (m^2^)	1.60	1.28	1.92	Fixed	NHSA's guidelines
Follow‐up treatment rate of TRAS + Chemo group (%)	0.47	0.00	1.00	Fixed	[13]
Follow‐up treatment rate of PEM + TRAS + Chemo group (%)	0.39	0.00	1.00	Fixed	[13]
Proportion of first‐line chemotherapy regimen selection					
The ratio of cisplatin and fluorouracil in the PEM + TRAS + Chemo group	0.15	—	—	Fixed	[13]
The ratio of cisplatin and fluorouracil in the TRAS + Chemo group	0.14	—	—	Fixed	[13]
The ratio of capecitabine and oxaliplatin in the PEM + TRAS + Chemo group	0.85	—	—	Fixed	[13]
The ratio of capecitabine and oxaliplatin in the TRAS + Chemo group	0.86	—	—	Fixed	[13]

Abbreviations: PD, progressive disease; PEM + TRAS + Chemo, pembrolizumab plus trastuzumab and chemotherapy; PFS, progression‐free survival; TRAS + Chemo, trastuzumab plus chemotherapy.

### 2.5 Uncertainty Analysis

To assess the robustness of the base‐case results, this study conducted one‐way deterministic sensitivity analysis (DSA), probabilistic sensitivity analysis (PSA) and scenario analysis. First, in the DSA, variations in the ICER were evaluated by varying input parameters within ±20% of their baseline values. The discount rate was tested separately across a range of 0%–8%. The results of this analysis were visually presented using a Tornado diagram. Second, the PSA performed 1000 iterations using Monte Carlo simulation, where cost and disutility parameters were modeled using the Gamma distribution, and clinical outcomes and health utility values were modeled using the Beta distribution. The outcomes of the PSA were presented as scatter plots and cost‐effectiveness acceptability curves (CEAC). Additionally, in the base‐case analysis utilizing a log‐logistic distribution for long‐term survival extrapolation, although the goodness‐of‐fit (assessed by AIC, BIC, and visual inspection) demonstrated statistical superiority over alternative models, minor discrepancies persisted when compared to the observed OS and PFS curves from clinical trials. To address this limitation, a scenario analysis employing a suboptimal parametric fit (lognormal distribution) was implemented to rigorously assess the robustness of the findings. This study evaluated cost‐effectiveness in accordance with the China Guidelines for Pharmacoeconomic Evaluations, setting the threshold at one to three times the per capita GDP per QALY [19]. According to officially reported data from the National Bureau of Statistics of China, the GDP per QALY in 2024 was calculated as $13,333.29 [29].

## 3 Results

### 3.1 Base‐Case and Subgroup Analysis

Patients receiving the PEM + TRAS + Chemo regimen achieved a QALY of 1.76 at a total cost of $76,914.57. In comparison, those treated with TRAS + Chemo alone attained a QALY of 1.58 at a total cost of $67,040.48. The ICER was calculated at $53,160.95 per QALY, exceeding three times China's per capita GDP ($39,999.86). A subgroup analysis of patients with a PD‐L1 CPS of ≥ 1 indicated that the PEM + TRAS + Chemo group gained an additional 0.31 QALYs, with an incremental cost of $15,525.22. Furthermore, the ICER remained above the threshold of three times China's per capita GDP. In China, the ICERs for both the overall population and subgroups exceeded three times the per capita GDP, suggesting that the PEM + TRAS + Chemo regimen may not be cost‐effective within the Chinese healthcare context. The results of the analysis are summarized in Table 2.

**TABLE 2:** Results of the base‐case and subgroup analysis.

Group	Base‐case analysis		Subgroup analysis (PD‐L1 CPS ≥ 1)	
	PEM + TRAS + Chemo group	TRAS + Chemo group	PEM + TRAS+Chemo group	TRAS + Chemo group
Cost				
PFS	42,126.59	17,346.73	42,763.67	17,017.51
PD	32,933.68	47,820.98	36,109.08	46,292.41
Death	1854.30	1872.77	1841.24	1878.85
Total	76,914.57	67,040.48	80,713.99	65,188.77
QALY				
PFS	1.21	0.90	1.24	0.88
PD	0.56	0.68	0.61	0.66
Total	1.76	1.58	1.85	1.54
LYs				
PFS	1.52	1.13	1.56	1.11
PD	0.96	1.18	1.05	1.14
Total	2.48	2.31	2.62	2.25
Incremental costs	9874.08	—	15,525.22	—
Incremental QALYs	0.19	—	0.31	—
Incremental LYs	0.17	—	0.37	—
ICER ($/QALYs)	53,160.95	—	49,849.43	—

*Note:* Incremental results are calculated relative to the TRAS + Chemo group.

Abbreviations: ICER, incremental cost‐effectiveness ratio; LY, life year; OS, overall survival; PD, progressive disease; PEM + TRAS + Chemo, pembrolizumab plus trastuzumab and chemotherapy; PFS, progression‐free survival; QALY, quality‐adjusted life year; TRAS + Chemo, trastuzumab plus chemotherapy.

### 3.2 Uncertainty Analysis

**FIGURE 3:**
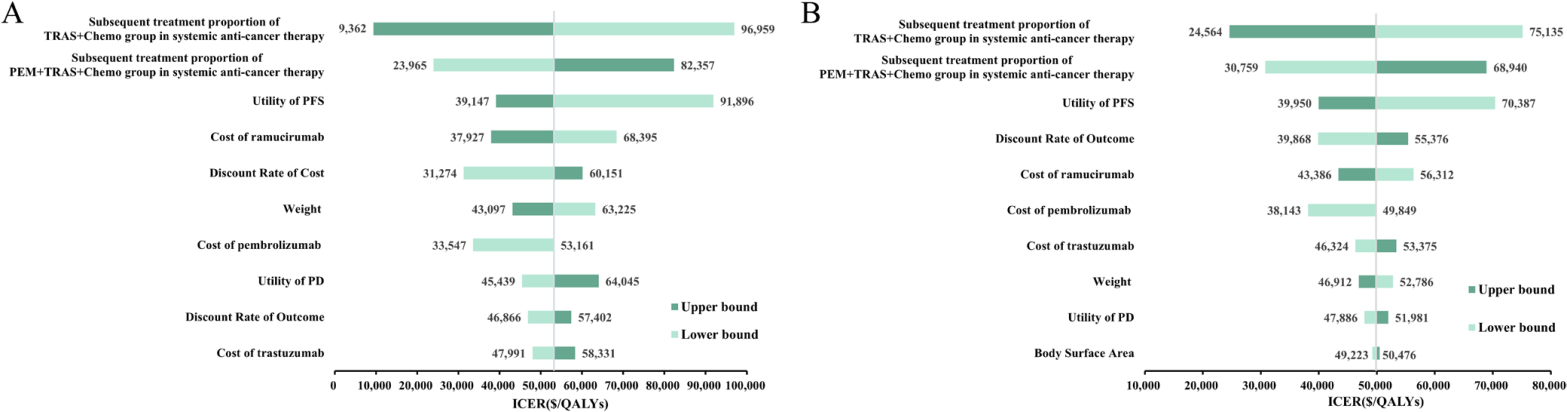
Tornado diagram of the DSA for all patients and patients with a PD‐L1 CPS ≥ 1. Tornado diagrams illustrating the results of the deterministic sensitivity analysis (DSA). (A) All patients. (B) Patients with a PD‐L1 combined positive score (CPS) ≥ 1. The figure shows the impact of varying key parameters on the incremental cost‐effectiveness ratio (ICER), where each bar represents the range of ICER values resulting from a ±20% variation in the corresponding parameter.

**FIGURE 4:**
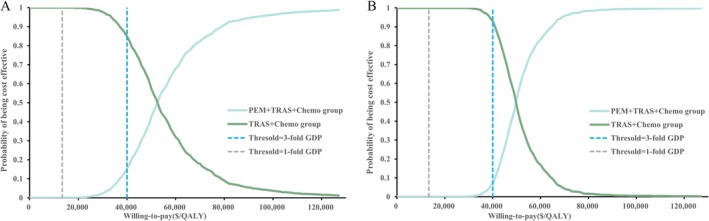
The cost‐effectiveness acceptability curve for all patients and patients with PD‐L1 CPS ≥ 1. Cost‐effectiveness acceptability curves (CEACs) illustrating the probability of pembrolizumab plus trastuzumab and chemotherapy being cost‐effective compared to trastuzumab and chemotherapy alone across a range of willingness‐to‐pay (WTP) thresholds. The curves are presented separately for (A) all patients and (B) patients with a PD‐L1 combined positive score (CPS) ≥ 1.

**FIGURE 5:**
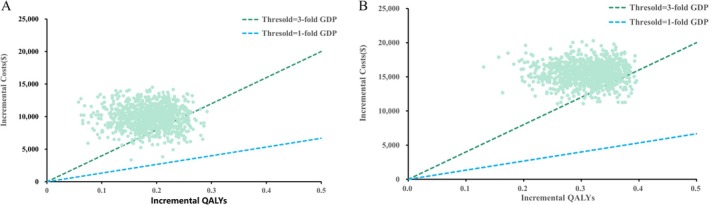
The cost‐effectiveness probabilistic scatter plot for all patients and patients with PD‐L1 CPS ≥ 1. (A) Cost‐effectiveness scatter plot for the total population. (B) Cost‐effectiveness scatter plot for patients with a PD‐L1 combined positive score (CPS) ≥ 1. Each point represents an incremental cost‐effectiveness ratio (ICER) estimate derived from 1000 Monte Carlo simulations.

This study assessed model robustness through comprehensive sensitivity analyses, with tornado diagrams illustrating the impact of key parameters on the ICER. Figure 3A shows that the proportion of patients receiving subsequent systemic therapy had the greatest impact on cost‐effectiveness. The utility of PFS ranked second, followed by the costs of ramucirumab. Figure 3B shows that, in patients with CPS ≥ 1, the proportion of subsequent therapy, PFS utility, and health outcome discount rate were the primary ICER determinants. The CEAC and probabilistic scatter plots are shown in Figures 4 and 5. According to the PSA, when benchmarked against a willingness‐to‐pay (WTP) threshold equivalent to three times the GDP per capita, the PEM + TRAS + Chemo group demonstrated a cost‐effectiveness probability of 15.16% across all patients and 7.08% among those with CPS ≥ 1. In the scenario analysis, replacing the log‐logistic distribution with the lognormal distribution resulted in an ICER of $52,503.85/QALY, corresponding to a 5.3% increase compared to the base‐case analysis. Under the alternative survival model, the probability of cost‐effectiveness at the WTP threshold of three times the GDP per capita was 14.27%. Full methodological details and associated results are presented in the Supporting Information.

## 4 Discussion

Therapeutic progress in HER2‐positive advanced gastric cancer has remained limited over the past decade [5]. In June 2024, China approved pembrolizumab in combination with trastuzumab and chemotherapy as a first‐line treatment for patients with locally advanced, unresectable, or metastatic HER2‐positive gastric or gastroesophageal junction adenocarcinoma, whose tumors express PD‐L1 CPS ≥ 1, as determined by a fully validated test. To our knowledge, this study is the first cost‐effectiveness analysis comparing the PEM + TRAS + Chemo regimen with the TRAS + Chemo regimen as a first‐line treatment for HER2‐positive gastric or GEJ adenocarcinoma.

Our parametric survival modeling results were highly consistent with the KEYNOTE‐811 trial data, with median OS and PFS nearly identical to the observed values and differences within 95% CIs. This consistency supports the reliability of the extrapolated survival curves and underpins the robustness of our cost‐effectiveness findings.

The base case analysis showed that, compared to the TRAS + Chemo regimen, the combination of PEM + TRAS + Chemo regimen increased QALYs by 0.19, but at an additional cost of $9874.08, resulting in an ICER of $53,160.95 per QALY. This ICER markedly exceeded China's WTP threshold, indicating that the PEM + TRAS + Chemo regimen is not cost‐effective in the current economic landscape. A subgroup analysis of patients with PD‐L1 CPS ≥ 1 was performed due to its demonstrated PFS and OS benefits. However, the results continued to indicate that the PEM + TRAS + Chemo regimen was not cost‐effective. From a health economics perspective, the observed survival benefits and quality‐of‐life improvements did not provide sufficient clinical value to justify the substantial additional costs. The scenario analysis demonstrated that substituting the survival model with a lognormal distribution caused only minor changes in the ICER (5.3% deviation) and did not alter the overall conclusion regarding cost‐ineffectiveness, with the probability of cost‐effectiveness remaining low at 14.27%. This reinforces the robustness of our findings despite uncertainties in long‐term survival extrapolation.

These findings align with previous economic evaluations, particularly Lang et al.'s Markov model analysis based on KEYNOTE‐859 trial data [30]. Their analysis estimated an ICER of $76,936.60/QALY for pembrolizumab‐chemotherapy, exceeding China's WTP threshold. Notably, in the PD‐L1 CPS ≥ 10 subgroup, the ICER declined to $34,813.70/QALY, positioning it below the cost‐effectiveness threshold. Similarly, Zheng et al. employed a PSM model and determined that the ICER for pembrolizumab in China was $177,405.83/QALY, exceeding the WTP threshold and indicating a lack of cost‐effectiveness at the standard price [31]. Lang et al. established cost‐effectiveness for CPS ≥ 10 patients in the US context, but this did not hold under China's economic benchmarks [25]. Although pembrolizumab provides survival benefits in advanced gastric cancer, its economic viability in China remains constrained, particularly in PD‐L1‐low populations. Despite its clinical benefits, the high cost of pembrolizumab limits its widespread adoption in China's healthcare system. For patients with high PD‐L1 expression, pembrolizumab may present a more cost‐effective treatment option; however, for the majority of patients, economic constraints hinder its widespread adoption in China.

However, this study's economic evaluation is based on data from the KEYNOTE‐811 Phase III clinical trial, which introduces several methodological limitations. The trial's limited Asian representation (34%), particularly the absence of Chinese subgroup stratification, constrains the generalizability of findings to China's healthcare setting. Additionally, there is a lack of large‐scale clinical trial data specifically focused on patients with gastric cancer in China. Furthermore, this study derived overall and progression‐free survival estimates through parametric distribution fitting, introducing uncertainty when extrapolating beyond the trial's follow‐up period. Moreover, while rigorous inclusion criteria ensured balanced baseline characteristics, real‐world variations in patient comorbidities, treatment adherence, and adverse events may differ significantly. Consequently, applying the findings of this clinical trial and our model analysis to real‐world patient populations remains challenging. In this study, most parameters, aside from drug costs, were sourced from existing literature, which may not fully capture the complexity of real‐world clinical scenarios.

## 5 Conclusion

While PEM + TRAS + Chemo regimen demonstrates clinical efficacy for HER2‐positive gastric/GEJ adenocarcinoma, its current cost‐effectiveness in China remains suboptimal versus TRAS + Chemo regimen. Future price negotiations or indication‐specific pricing for pembrolizumab could influence future cost‐effectiveness assessments.

## Author Contributions

**Yifang Liang:** took the lead in conceptualization, data curation, investigation, software, methodology, and writing – original draft. Contributed equally to formal analysis, validation, and visualization. **Yuyanzi Zhang:** contributed substantially to data curation, formal analysis, and methodology. Participated in writing – original draft. **Hongfei Hu:** participated in data collection, investigation, and formal analysis. Contributed to software‐related tasks and resource organization. **Yan Li:** supported data processing and validation. Assisted in the review and editing process. **Aixia Ma:** served as co‐corresponding author. Led supervision and project coordination. Contributed to conceptualization and writing – review and editing. **Xin Guan:** served as co‐corresponding author. Played a leading role in supervision, conceptualization, and writing – review and editing. Also contributed to validation and visualization. All authors have reviewed and approved the final version of the manuscript.

## Ethics Statement

The authors have nothing to report.

## Conflicts of Interest

The authors declare no conflicts of interest.

## Supporting information


**Data S1:** cam471379‐sup‐0001‐supinfo.docx.

## Data Availability

The data used in this study were obtained from the KEYNOTE‐811 trial and published literature. All data sources are publicly available and cited within the manuscript. Additional model inputs are available from the corresponding author upon reasonable request.
