# Supplementary material for: Cost‐Effectiveness of Pembrolizumab Plus Trastuzumab and Chemotherapy Versus Trastuzumab Plus Chemotherapy as First‐Line Treatment of HER2‐Positive Gastric or Gastroesophageal Junction Adenocarcinoma in China
Source: Cancer Med. 2025 Nov 20;14(22):e71379. doi: 10.1002/cam4.71379 (PMC12631738; doi:10.1002/cam4.71379)
Supplement: Supplementary file 1 — Data S1: cam471379‐sup‐0001‐supinfo.docx. [file CAM4-14-e71379-s001.docx]

Cost-effectiveness of pembrolizumab plus trastuzumab and chemotherapy vs trastuzumab plus chemotherapy as first-line treatment of HER2-positive gastric or gastroesophageal junction adenocarcinoma in China

SUPPLEMENTS

Contents

[Supplementary Figure 1. Fitting and extrapolation of Kaplan Meier survival curve for overall patients. 1](#_Toc196749662)

[Supplementary Figure 2. Fitting and extrapolation of Kaplan Meier survival curve for patients with PD-L1 CPS≥1. 2](#_Toc196749663)

[Supplementary Figure 3. Tornado diagram of the deterministic sensitivity analysis for the scenario analysis. 3](#_Toc196749664)

[Supplementary Figure 4. Cost-effectiveness acceptability curve from the scenario analysis. 3](#_Toc196749665)

[Supplementary Table 1. Baseline characteristics of patients in KEYNOTE-811 4](#_Toc196749706)

[Supplementary Table 2. The Akaike information criteria and Bayesian information criteria 5](#_Toc196749707)

[Supplementary Table 3. The akaike information criteria and Bayesian information criteria (PD-L1 CPS ≥ 1). 5](#_Toc196749708)

[Supplementary Table 4. Estimated Model Parameters for OS (Total Population, PEM+TRAS+Chemo Group) 5](#_Toc196749709)

[Supplementary Table 5. Estimated Model Parameters for PFS (Total Population, PEM+TRAS+Chemo Group) 6](#_Toc196749710)

[Supplementary Table 6. Estimated Model Parameters for OS (Total Population, TRAS+Chemo Group) 6](#_Toc196749711)

[Supplementary Table 7. Estimated Model Parameters for PFS (Total Population, TRAS+Chemo Group) 6](#_Toc196749712)

[Supplementary Table 8.Estimated Model Parameters for OS (PD-L1 CPS ≥ 1 Subgroup, PEM+TRAS+ Chemo Group) 7](#_Toc196749713)

[Supplementary Table 9. Estimated Model Parameters for PFS (PD-L1 CPS ≥ 1 Subgroup, PEM+TRAS + Chemo Group) 7](#_Toc196749714)

[Supplementary Table 10. Estimated Model Parameters for OS (PD-L1 CPS ≥ 1 Subgroup, TRAS+Chemo Group) 7](#_Toc196749715)

[Supplementary Table 11. Estimated Model Parameters for PFS (PD-L1 CPS ≥ 1 Subgroup, TRAS+Chemo Group) 8](#_Toc196749716)

[Supplementary Table 12. Results of the scenario analysis based on the lognormal survival distribution. 8](#_Toc196749717)

[Supplementary Table 13. CHEERS Checklist (2022) 9](#_Toc196749718)


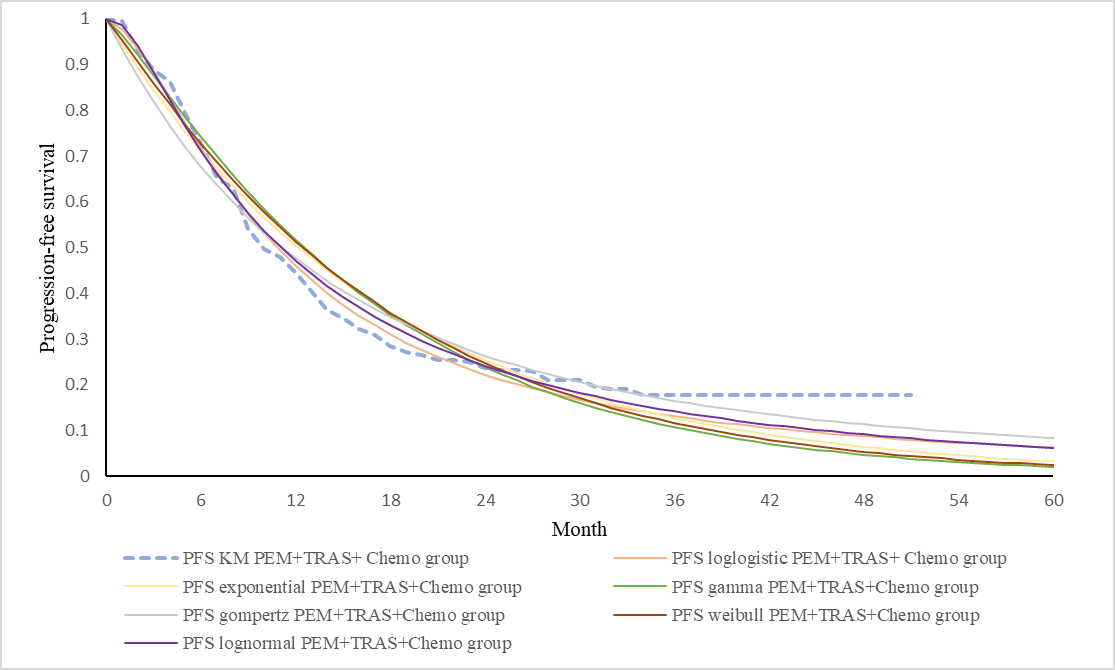

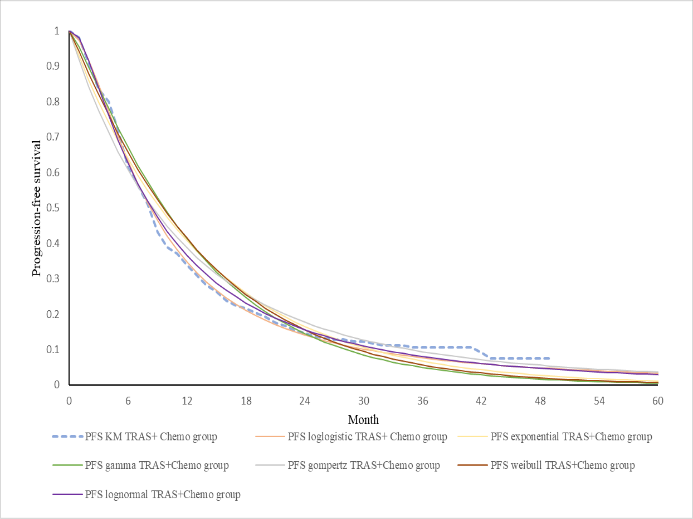

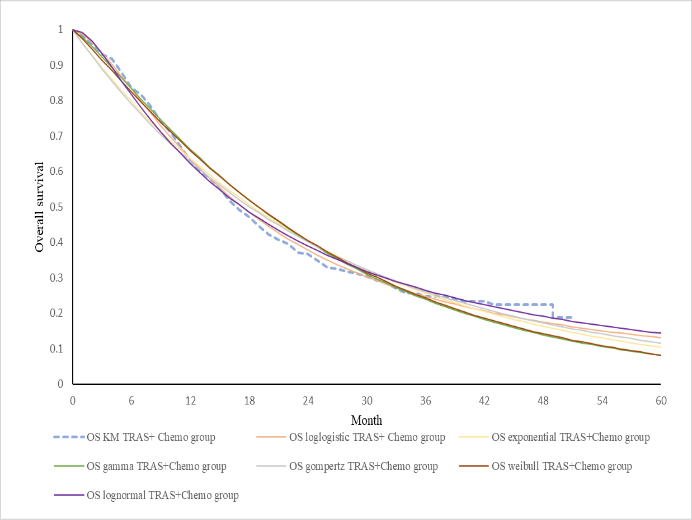

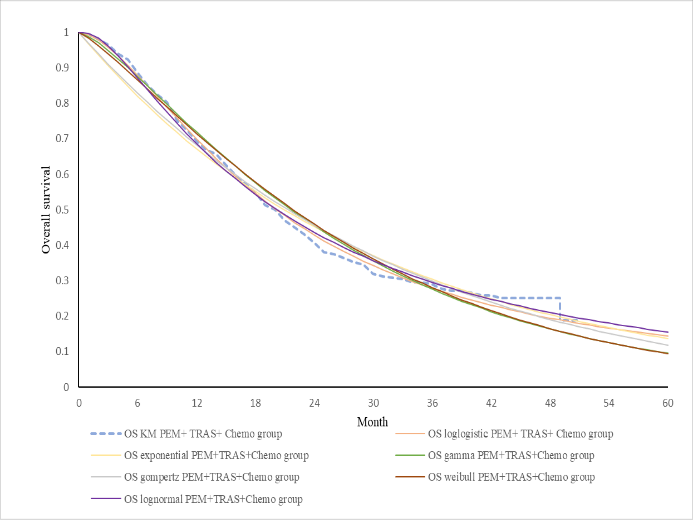


(d)

(c)

(b)

(a)

**Supplementary Figure 1. Fitting and extrapolation of Kaplan Meier survival curve for overall patients.** (a) The results of OS curve in PEM + TRAS + Chemo arm. (b) The results of PFS curve in PEM + TRAS + Chemo arm. (c) The results of OS curve in TRAS + Chemo arm. (d) The results of PFS curve in TRAS + Chemo arm. PEM + TRAS + Chemo = pembrolizumab plus trastuzumab and chemotherapy; TRAS + Chemo = trastuzumab plus chemotherapy; OS = overall survival; PFS = progression-free survival.


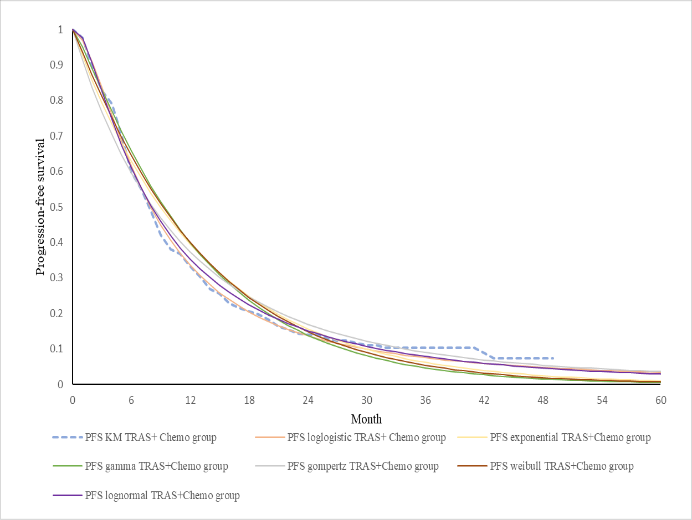

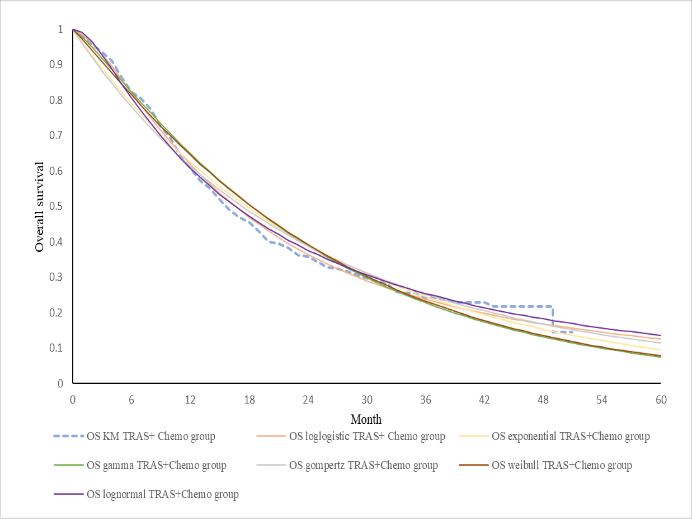

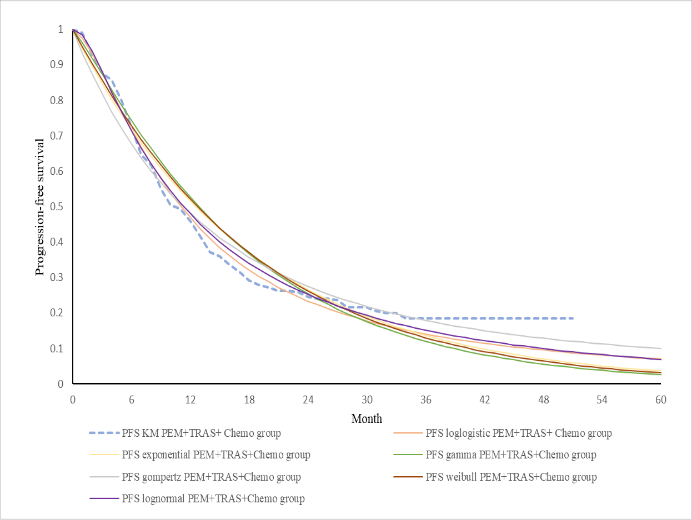

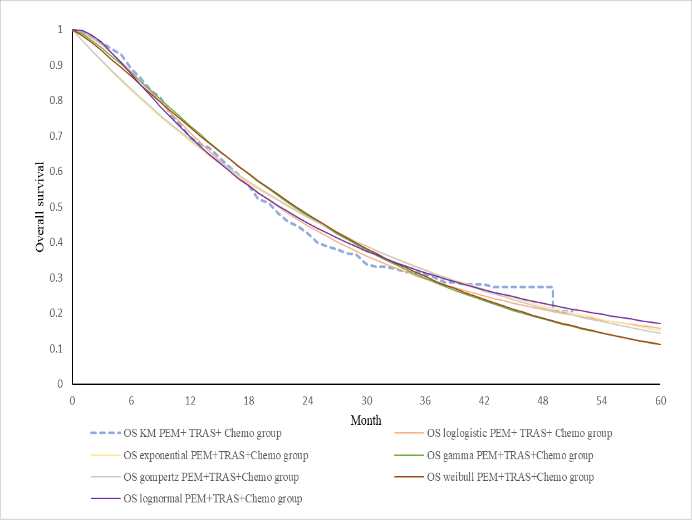


(d)

(b)

(a)

(c)

**Supplementary Figure 2. Fitting and extrapolation of Kaplan Meier survival curve for patients with PD-L1 CPS≥1.** (a) The results of OS curve in PEM + TRAS + Chemo arm. (b) The results of PFS curve in PEM + TRAS + Chemo arm. (c) The results of OS curve in TRAS + Chemo arm. (d) The results of PFS curve in TRAS + Chemo arm. PEM + TRAS + Chemo = pembrolizumab plus trastuzumab and chemotherapy; TRAS + Chemo = trastuzumab plus chemotherapy; OS = overall survival; PFS = progression-free survival.


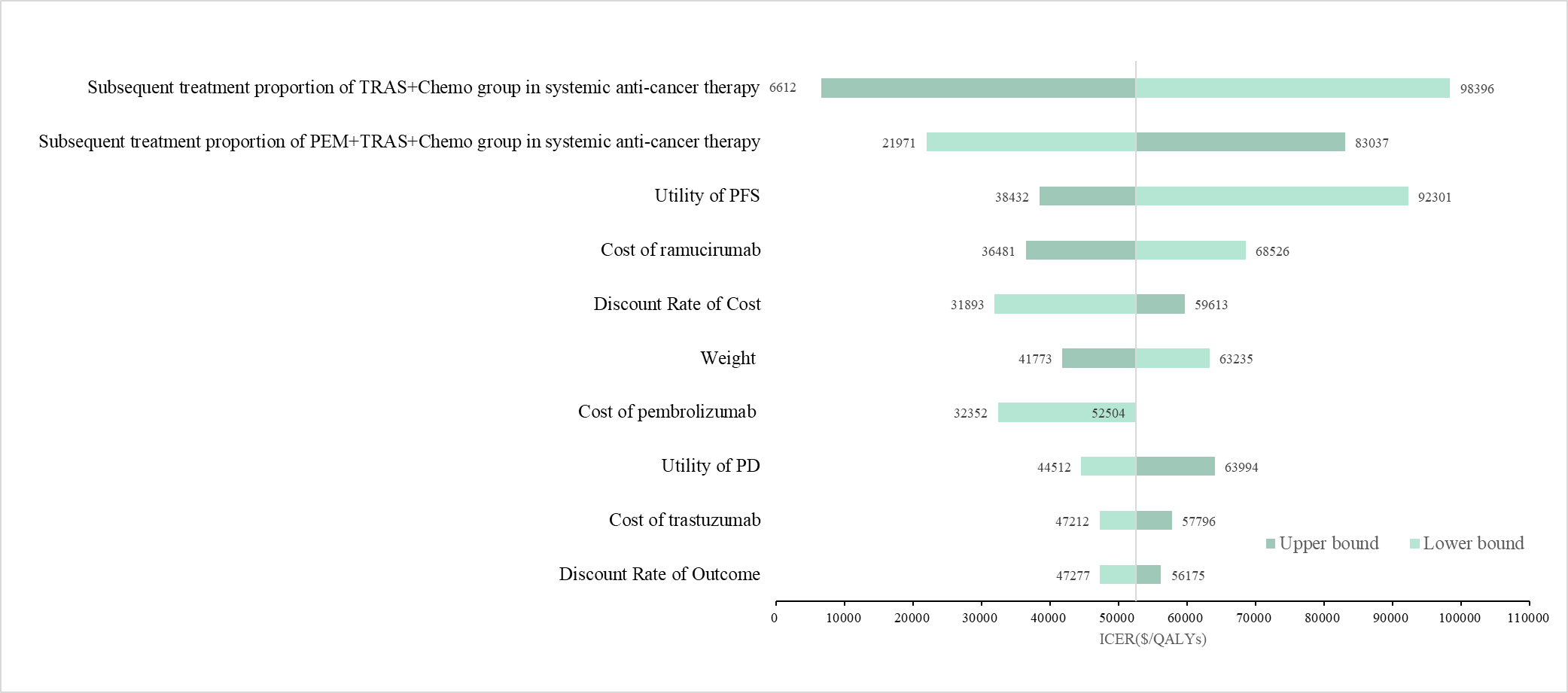


**Supplementary Figure 3. Tornado diagram of the deterministic sensitivity analysis for the scenario analysis.**


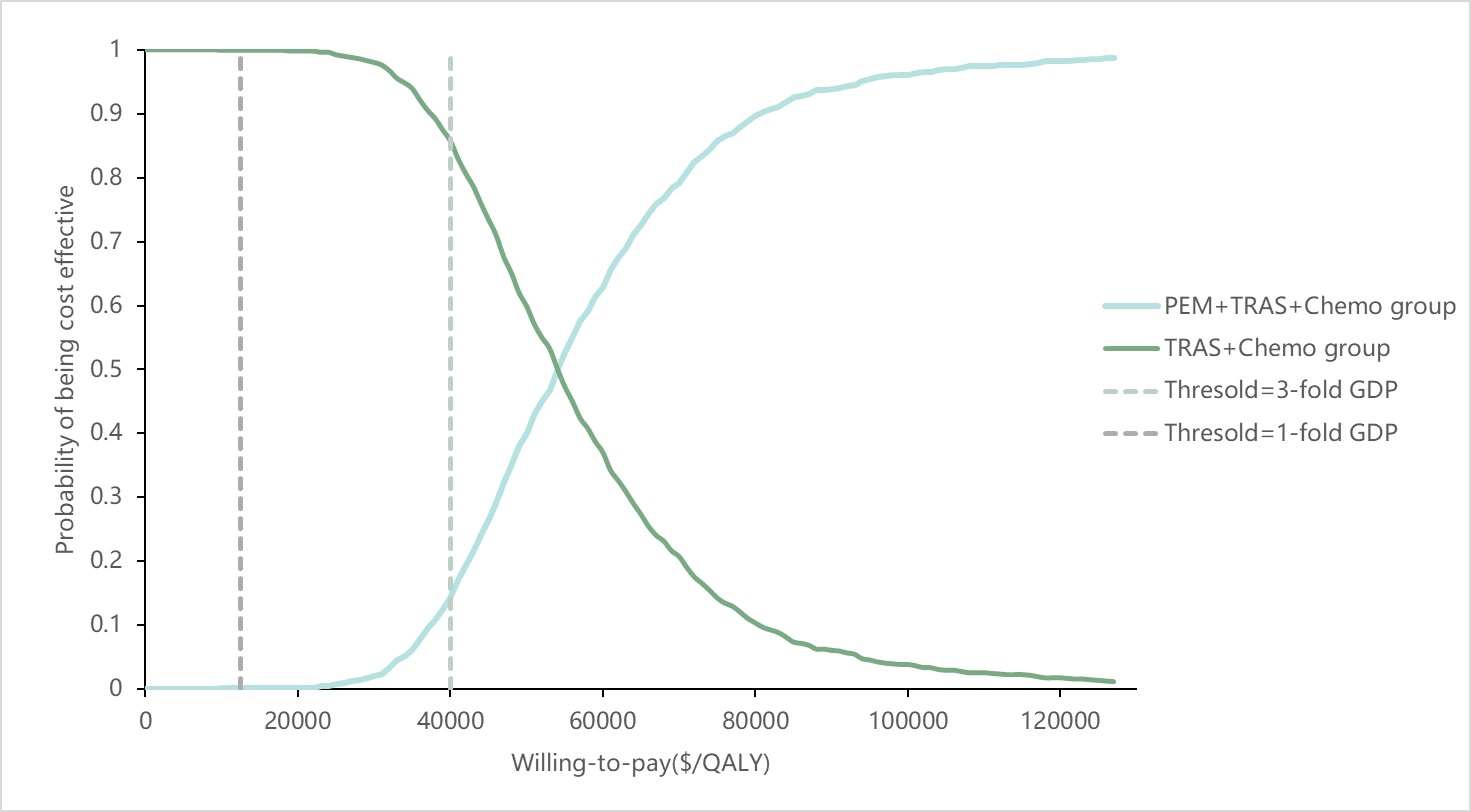


**Supplementary Figure 4. Cost-effectiveness acceptability curve from the scenario analysis.**

**Supplementary Table 1. Baseline characteristics of patients in KEYNOTE-811.**

| Characteristic | Pembrolizumab (N=350) | Placebo (N=348) |
| --- | --- | --- |
| Age, years | 62 (54–69) | 63 (55–70) |
| ≥65 years | 145 (41%) | 156 (45%) |
| Sex |  |  |
| Female | 66 (19%) | 68 (20%) |
| Male | 284 (81%) | 280 (80%) |
| Race |  |  |
| American Indian or Native Alaskan | 5 (1%) | 6 (2%) |
| Asian | 119 (34%) | 121 (35%) |
| Black or African American | 2 (1%) | 2 (1%) |
| Multiple | 6 (2%) | 5 (1%) |
| White | 218 (62%) | 212 (61%) |
| Missing | 0 | 2 (1%) |
| Geographical region |  |  |
| Australia, Europe, Israel, North America | 113 (32%) | 111 (32%) |
| Asia | 118 (34%) | 119 (34%) |
| Rest of world | 119 (34%) | 118 (34%) |
| PD-L1 status |  |  |
| CPS ≥1 | 298 (85%) | 296 (85%) |
| CPS <1 | 52 (15%) | 52 (15%) |
| Chemotherapy regimen |  |  |
| Capecitabine and oxaliplatin | 297 (85%) | 299 (86%) |
| Fluorouracil and cisplatin | 53 (15%) | 49 (14%) |

Note: This table was extracted from the KEYNOTE-811. Content of this table reflected the baseline characteristics of patients with HER2-positive gastric or gastro-oesophageal junction adenocarcinoma in the KEYNOTE-811 trial. CPS= combined positive score.

**Supplementary Table 2. The Akaike information criteria and Bayesian information criteria.**

|  | PEM+ TRAS  + Chemo group  OS | | TRAS+ Chemo  group  OS | | PEM+ TRAS  + Chemo group  PFS | | TRAS+ Chemo  group  PFS | |
| --- | --- | --- | --- | --- | --- | --- | --- | --- |
| Distribution | AIC | BIC | AIC | BIC | AIC | BIC | AIC | BIC |
| Exponential | 2162.10 | 2165.96 | 2191.25 | 2195.11 | 1957.09 | 1960.94 | 1877.20 | 1881.06 |
| Gamma | 2147.45 | 2155.17 | 2186.42 | 2194.12 | 1953.27 | 1960.99 | 1871.05 | 1878.75 |
| Gompertz | 2163.50 | 2171.21 | 2192.92 | 2200.62 | 1951.81 | 1959.53 | 1873.23 | 1880.93 |
| Weibull | 2152.39 | 2160.11 | 2189.39 | 2197.10 | 1957.61 | 1965.33 | 1876.93 | 1884.64 |
| Loglogistic | 2133.37 | 2141.08 | 2170.81 | 2178.51 | 1915.80 | 1923.51 | 1829.47 | 1837.17 |
| Lognormal | 2137.18 | 2144.90 | 2178.93 | 2186.64 | 1915.94 | 1923.66 | 1832.33 | 1840.03 |

Note: PEM + TRAS + Chemo = pembrolizumab plus trastuzumab and chemotherapy; TRAS + Chemo = trastuzumab plus chemotherapy; OS = overall survival; PFS = progression-free survival; AIC=Akaike information criterion; BIC= Bayesian information criterion.

**Supplementary Table 3. The akaike information criteria and Bayesian information criteria (PD-L1 CPS ≥ 1).**

|  | PEM+ TRAS  + Chemo group  OS | | TRAS+ Chemo  group  OS | | PEM+ TRAS  +Chemo group  PFS | | TRAS+ Chemo  group  PFS | |
| --- | --- | --- | --- | --- | --- | --- | --- | --- |
| Distribution | AIC | BIC | AIC | BIC | AIC | BIC | AIC | BIC |
| Exponential | 1821.88 | 1825.57 | 1860.79 | 1864.48 | 1677.91 | 1681.61 | 1597.18 | 1600.87 |
| Gamma | 1812.01 | 1819.41 | 1857.86 | 1865.24 | 1676.69 | 1684.08 | 1593.63 | 1601.02 |
| Gompertz | 1823.78 | 1831.18 | 1862.15 | 1869.53 | 1671.22 | 1678.62 | 1593.07 | 1600.46 |
| Weibull | 1815.95 | 1823.35 | 1860.29 | 1867.67 | 1679.46 | 1686.85 | 1597.91 | 1605.29 |
| Loglogistic | 1798.92 | 1806.32 | 1843.32 | 1850.70 | 1643.56 | 1650.96 | 1558.86 | 1566.24 |
| Lognormal | 1801.61 | 1809.00 | 1848.57 | 1855.95 | 1642.02 | 1649.41 | 1561.85 | 1569.23 |

Note: PEM + TRAS + Chemo = pembrolizumab plus trastuzumab and chemotherapy; TRAS + Chemo = trastuzumab plus chemotherapy; PD-L1 CPS ≥1= PD-L1 combined positive score of 1 or higher ;OS = overall survival; PFS = progression-free survival; AIC=Akaike information criterion; BIC= Bayesian information criterion.

**Supplementary Table 4. Estimated Model Parameters for OS (Total Population, PEM + TRAS + Chemo Group).**

| **Distribution** | **AIC** | **BIC** | **Parameters** |
| --- | --- | --- | --- |
| Exponential | 2162.10 | 2165.96 | rate = 0.033 (SE: 0.002) |
| Gamma | 2147.45 | 2155.17 | shape = 1.411 (SE: 0.114); rate = 0.051 (SE: 0.006) |
| Gompertz | 2163.50 | 2171.21 | shape = 0.004 (SE: 0.006); rate = 0.031 (SE: 0.003) |
| Weibull | 2152.39 | 2160.11 | shape = 1.213 (SE: 0.066); scale = 29.544 (SE: 1.560) |
| Log-logistic | 2133.37 | 2141.08 | shape = 1.636 (SE: 0.088); scale = 20.144 (SE: 1.167) |
| Log-normal | 2137.18 | 2144.90 | meanlog = 3.006 (SE: 0.061); sdlog = 1.074 (SE: 0.052) |

**Supplementary Table 5. Estimated Model Parameters for PFS (Total Population, PEM + TRAS + Chemo Group).**

| **Distribution** | **AIC** | **BIC** | **Parameters** |
| --- | --- | --- | --- |
| Exponential | 1957.09 | 1960.94 | rate = 0.057 (SE: 0.004) |
| Gamma | 1953.27 | 1960.99 | shape = 1.211 (SE: 0.094); rate = 0.072 (SE: 0.008) |
| Gompertz | 1951.81 | 1959.53 | shape = -0.019 (SE: 0.007); rate = 0.069 (SE: 0.006) |
| Weibull | 1957.61 | 1965.33 | shape = 1.064 (SE: 0.053); scale = 17.551 (SE: 1.038) |
| Log-logistic | 1915.80 | 1923.51 | shape = 1.575 (SE: 0.082); scale = 10.840 (SE: 0.665) |
| Log-normal | 1915.94 | 1923.66 | meanlog = 2.405 (SE: 0.063); sdlog = 1.101 (SE: 0.051) |

**Supplementary Table 6. Estimated Model Parameters for OS (Total Population, TRAS + Chemo Group).**

| **Distribution** | **AIC** | **BIC** | **Parameters** |
| --- | --- | --- | --- |
| Exponential | 2191.25 | 2195.11 | rate = 0.038 (SE: 0.002) |
| Gamma | 2186.42 | 2194.12 | shape = 1.234 (SE: 0.097); rate = 0.049 (SE: 0.005) |
| Gompertz | 2192.92 | 2200.62 | shape = -0.003 (SE: 0.006); rate = 0.040 (SE: 0.004) |
| Weibull | 2189.39 | 2197.10 | shape = 1.112 (SE: 0.059); scale = 26.323 (SE: 1.480) |
| Log-logistic | 2170.81 | 2178.51 | shape = 1.520 (SE: 0.080); scale = 17.298 (SE: 1.069) |
| Log-normal | 2178.93 | 2186.64 | meanlog = 2.849 (SE: 0.066); sdlog = 1.169 (SE: 0.055) |

**Supplementary Table 7. Estimated Model Parameters for PFS (Total Population, TRAS + Chemo Group).**

| **Distribution** | **AIC** | **BIC** | **Parameters** |
| --- | --- | --- | --- |
| Exponential | 1877.20 | 1881.06 | rate = 0.075 (SE: 0.005) |
| Gamma | 1871.05 | 1878.75 | shape = 1.247 (SE: 0.094); rate = 0.096 (SE: 0.010) |
| Gompertz | 1873.23 | 1880.93 | shape = -0.017 (SE: 0.007); rate = 0.087 (SE: 0.008) |
| Weibull | 1876.93 | 1884.64 | shape = 1.074 (SE: 0.050); scale = 13.489 (SE: 0.783) |
| Log-logistic | 1829.47 | 1837.17 | shape = 1.692 (SE: 0.086); scale = 8.258 (SE: 0.479) |
| Log-normal | 1832.33 | 1840.03 | meanlog = 2.128 (SE: 0.060); sdlog = 1.040 (SE: 0.046) |

**Supplementary Table 8. Estimated Model Parameters for OS (PD-L1 CPS ≥ 1 Subgroup, PEM + TRAS + Chemo Group).**

| **Distribution** | **AIC** | **BIC** | **Parameters** |
| --- | --- | --- | --- |
| Exponential | 1821.88 | 1825.57 | rate = 0.031 (SE: 0.027) |
| Gamma | 1812.01 | 1819.41 | shape = 1.371 (SE: 1.152);  rate = 0.047 (SE: 0.037) |
| Gompertz | 1823.78 | 1831.18 | shape = 0.002 (SE: 0.010);  rate = 0.031 (SE: 0.024) |
| Weibull | 1815.95 | 1823.35 | shape = 1.190 (SE: 0.071);  scale = 31.063 (SE: 1.837) |
| Log-logistic | 1798.92 | 1806.32 | shape = 1.597 (SE: 0.095);  scale = 21.029 (SE: 1.358) |
| Log-normal | 1801.61 | 1809.00 | meanlog = 3.054 (SE: 0.068);  sdlog = 1.098 (SE: 0.058) |

**Supplementary Table 9. Estimated Model Parameters for PFS (PD-L1 CPS ≥ 1 Subgroup, PEM + TRAS + Chemo Group).**

| **Distribution** | **AIC** | **BIC** | **Parameters** |
| --- | --- | --- | --- |
| Exponential | 1677.91 | 1681.61 | rate = 0.055 (SE: 0.004) |
| Gamma | 1676.69 | 1684.08 | shape = 1.166 (SE: 0.098);  rate = 0.066 (SE: 0.008) |
| Gompertz | 1671.22 | 1678.62 | shape = -0.022 (SE: 0.008);  rate = 0.069 (SE: 0.007) |
| Weibull | 1679.46 | 1686.85 | shape = 1.038 (SE: 0.057);  scale = 18.141 (SE: 1.192) |
| Log-logistic | 1643.56 | 1650.96 | shape = 1.530 (SE: 0.087);  scale = 11.055 (SE: 0.756) |
| Log-normal | 1642.02 | 1649.41 | meanlog = 2.428 (SE: 0.070);  sdlog = 1.126 (SE: 0.057) |

**Supplementary Table 10. Estimated Model Parameters for OS (PD-L1 CPS ≥ 1 Subgroup, TRAS + Chemo Group).**

| **Distribution** | **AIC** | **BIC** | **Parameters** |
| --- | --- | --- | --- |
| Exponential | 1860.79 | 1864.48 | rate = 0.039 (SE: 0.003) |
| Gamma | 1857.86 | 1865.24 | shape = 1.212 (SE: 0.103);  rate = 0.049 (SE: 0.006) |
| Gompertz | 1862.15 | 1869.53 | shape = -0.005 (SE: 0.006);  rate = 0.042 (SE: 0.005) |
| Weibull | 1860.29 | 1867.67 | shape = 1.096 (SE: 0.062);  scale = 25.536 (SE: 1.574) |
| Log-logistic | 1843.32 | 1850.70 | shape = 1.505 (SE: 0.086);  scale = 16.607 (SE: 1.124) |
| Log-normal | 1848.57 | 1855.95 | meanlog = 2.810 (SE: 0.072);  sdlog = 1.173 (SE: 0.059) |

**Supplementary Table 11. Estimated Model Parameters for PFS (PD-L1 CPS ≥ 1 Subgroup, TRAS + Chemo Group).**

| **Distribution** | **AIC** | **BIC** | **Parameters** |
| --- | --- | --- | --- |
| Exponential | 1597.18 | 1600.87 | rate = 0.077 (SE: 0.005) |
| Gamma | 1593.63 | 1601.02 | shape = 1.216 (SE: 0.098);  rate = 0.097 (SE: 0.011) |
| Gompertz | 1593.07 | 1600.46 | shape = -0.018 (SE: 0.008);  rate = 0.091 (SE: 0.008) |
| Weibull | 1597.91 | 1605.29 | shape = 1.059 (SE: 0.053);  scale = 13.054 (SE: 0.831) |
| Log-logistic | 1558.86 | 1566.24 | shape = 1.667 (SE: 0.092);  scale = 7.964 (SE: 0.507) |
| Log-normal | 1561.85 | 1569.23 | meanlog = 2.088 (SE: 0.066);  sdlog = 1.058 (SE: 0.051) |

**Supplementary Table 12. Results of the scenario analysis based on the lognormal survival distribution.**

| Group | Scenario analysis analysis | |
| --- | --- | --- |
|  | PEMB +TRAS +Chemo group | TRAS +Chemo group |
| **Cost** |  |  |
| PFS | 41,863.40 | 17,072.38 |
| PD | 33,571.92 | 48,840.11 |
| Death | 1,857.38 | 1,874.63 |
| Total | 77,292.69 | 67,787.12 |
| **QALY** |  |  |
| PFS | 1.19 | 0.88 |
| PD | 0.57 | 0.70 |
| Total | 1.76 | 1.58 |
| **LYs** |  |  |
| PFS | 1.50 | 1.11 |
| PD | 0.98 | 1.21 |
| Total | 2.48 | 2.32 |
| Incremental Costs | 9,505.58 | - |
| Incremental QALYs | 0.18 | - |
| Incremental LYs | 0.17 | - |
| ICER($/QALYs) | 52,503.85 | - |

Note: PEM + TRAS + Chemo = pembrolizumab plus trastuzumab and chemotherapy; TRAS + Chemo = trastuzumab plus chemotherapy; OS = overall survival; PFS = progression-free survival; PD = progressive disease; QALY = quality-adjusted life year; LY = life year; ICER = incremental cost-effectiveness ratio.

Incremental results are calculated relative to the TRAS + Chemo group.

**Supplementary Table 13. CHEERS Checklist (2022).**

| **Topic** | **No.** | **Item** | **Location where items is reported** |
| --- | --- | --- | --- |
| **Title** |  |  |  |
|  | 1 | Identify the study as an economic evaluation and specify the interventions being compared. | Section "Title page" |
| **Abstract** |  |  |  |
|  | 2 | Provide a structured summary that highlights context, key methods, results, and alternative analyses. | Section "Abstract" |
| **Introduction** |  |  |  |
| Background and objectives | 3 | Give the context for the study, the study question, and its practical relevance for decision making in policy or practice. | Section "Introduction" |
| **Methods** |  |  |  |
| Health economic analysis plan | 4 | Indicate whether a health economic analysis plan was developed and where available. | Not applicable |
| Study population | 5 | Describe characteristics of the study population (such as age range, demographics, socioeconomic, or clinical characteristics). | Section "Population and Interventions"; Supplementary Table 1 |
| Setting and location | 6 | Provide relevant contextual information that may influence findings. | Section "Population and Interventions"; |
| Comparators | 7 | Describe the interventions or strategies being compared and why chosen. | Section "Population and Interventions"; |
| Perspective | 8 | State the perspective(s) adopted by the study and why chosen. | Section "Inputs of cost and utility" |
| Time horizon | 9 | State the time horizon for the study and why appropriate. | Section "Model structure" |
| Discount rate | 10 | Report the discount rate(s) and reason chosen. | Section "Model structure" |
| Selection of outcomes | 11 | Describe what outcomes were used as the measure(s) of benefit(s) and harm(s). | Section "Model structure" |
| Measurement of outcomes | 12 | Describe how outcomes used to capture benefit(s) and harm(s) were measured. | Section "Sensitivity analysis" and "Inputs of cost and utility" |
| Valuation of outcomes | 13 | Describe the population and methods used to measure and value outcomes. | Section "Sensitivity analysis" |
| Measurement and valuation of resources and costs | 14 | Describe how costs were valued. | Section "Inputs of cost and utility" |
| Currency, price date, and conversion | 15 | Report the dates of the estimated resource quantities and unit costs, plus the currency and year of conversion. | Section "Model structure" |
| Rationale and description of model | 16 | If modelling is used, describe in detail and why used. Report if the model is publicly available and where it can be accessed. | Section "Model structure" |
| Analytics and assumptions | 17 | Describe any methods for analysing or statistically transforming data, any extrapolation methods, and approaches for validating any model used. | Section "Model structure" |
| Characterising heterogeneity | 18 | Describe any methods used for estimating how the results of the study vary for subgroups. | Section "Base-Case and Subgroup Analysis" |
| Characterising distributional effects | 19 | Describe how impacts are distributed across different individuals or adjustments made to reflect priority populations. | Not applicable |
| Characterising uncertainty | 20 | Describe methods to characterise any sources of uncertainty in the analysis. | Section "Sensitivity analysis" |
| Approach to engagement with patients and others affected by the study | 21 | Describe any approaches to engage patients or service recipients, the general public, communities, or stakeholders (such as clinicians or payers) in the design of the study. | Not applicable |
| **Results** |  |  |  |
| Study parameters | 22 | Report all analytic inputs (such as values, ranges, references) including uncertainty or distributional assumptions. | Section "Sensitivity analysis" |
| Summary of main results | 23 | Report the mean values for the main categories of costs and outcomes of interest and summarise them in the most appropriate overall measure. | Section "Base-Case and Subgroup Analysis" |
| Effect of uncertainty | 24 | Describe how uncertainty about analytic judgments, inputs, or projections affect findings. Report the effect of choice of discount rate and time horizon, if applicable. | Section "Sensitivity analysis" |
| Effect of engagement with patients and others affected by the study | 25 | Report on any difference patient/service recipient, general public, community, or stakeholder involvement made to the approach or findings of the study | Not applicable |
| **Discussion** |  |  |  |
| Study findings, limitations, generalisability, and current knowledge | 26 | Report key findings, limitations, ethical or equity considerations not captured, and how these could affect patients, policy, or practice. | Section "Discussion" |
| **Other relevant information** |  |  |  |
| Source of funding | 27 | Describe how the study was funded and any role of the funder in the identification, design, conduct, and reporting of the analysis | Section "Funding" |
| Conflicts of interest | 28 | Report authors conflicts of interest according to journal or International Committee of Medical Journal Editors requirements. | Section "Conflicts of interest" |

From: Husereau, Don et al. “Consolidated Health Economic Evaluation Reporting Standards (CHEERS) 2022 Explanation and Elaboration: A Report of the ISPOR CHEERS II Good Practices Task Force.” Value in health: the journal of the International Society for Pharmacoeconomics and Outcomes Research vol. 25,1 (2022): 10-31. doi:10.1016/j.jval.2021.10.008
